# Next Stage Approach to Tissue Engineering Skeletal Muscle

**DOI:** 10.3390/bioengineering7040118

**Published:** 2020-09-30

**Authors:** Gregory Reid, Fabio Magarotto, Anna Marsano, Michela Pozzobon

**Affiliations:** 1 Department of Cardiac Surgery, University Hospital Basel, 4031 Basel, Switzerland; gregory.reid@unibas.ch (G.R.); anna.marsano@usb.ch (A.M.); 2 Department of Biomedicine, University of Basel, 4031 Basel, Switzerland; 3 Department of Women’s and Children’s Health, University of Padova, 35128 Padova, Italy; fabio.magarotto.2@gmail.com; 4 Institute of Pediatric Research, Città della Speranza, 35127 Padova, Italy

**Keywords:** skeletal muscle engineering, neuro-angiogenesis, growth factors, extracellular vesicles

## Abstract

Large-scale muscle injury in humans initiates a complex regeneration process, as not only the muscular, but also the vascular and neuro-muscular compartments have to be repaired. Conventional therapeutic strategies often fall short of reaching the desired functional outcome, due to the inherent complexity of natural skeletal muscle. Tissue engineering offers a promising alternative treatment strategy, aiming to achieve an engineered tissue close to natural tissue composition and function, able to induce long-term, functional regeneration after in vivo implantation. This review aims to summarize the latest approaches of tissue engineering skeletal muscle, with specific attention toward fabrication, neuro-angiogenesis, multicellularity and the biochemical cues that adjuvate the regeneration process.

## 1. Introduction

Volumetric muscle loss (VML) occurs after an abrupt loss of skeletal muscle, often due to surgical resection, trauma or burns. There is currently no exact definition for VML, yet when a critical level of muscle loss is surpassed, a functional recovery is no longer possible. Scar tissue is deposited mostly by fibroblasts and an array of pathways lead the attempted regeneration [1]. The quantitative threshold of how much muscle loss can spontaneously be recovered post-injury is open to debate and also seems location (muscle) and species dependent, with the limit having been reported, for example, at 15% in mice [2] and 30% in rats [3], while also leading to a dysregulation of the neuromotor and fibrotic response. The severity of the disease is further enhanced by associated cosmetic and psychosocial problems often present. Lengthy treatment follow-ups and recurring operations lead to a high rate of patient dissatisfaction accompanied by the traumatic and stressful burden of an impaired everyday life.

Conventional therapeutic strategies often fail to produce the expected results. As an example, autologous muscle flaps, the current standard, are associated with varying transplant survival and donor site morbidity, also requiring a highly skilled surgeon. Post-operative treatment is of a physiotherapeutic nature and can only recover a certain level of functionality.

Tissue engineering offers an exciting alternative to overcome these limitations, the ultimate goal being off-the-shelf, patient-specific muscle grafts. The late 1990s brought an array of breakthroughs, [4,5], setting the foundation for current technologies in this field.

As a relatively simple alternative to traditional plastic surgery, acellular scaffolds implanted into a VML defect provide a cost-effective solution. They rely on host-cell invasion and incorporation into the host muscle system. The results found in the literature, however, fail to demonstrate a decisive and reproducible functional recovery [6,7]. This strategy relies on the host recipient for final functional maturation and is often readily vascularized, albeit without adequate myogenic cell invasion. A natural continuation of this concept, engineering a scaffold containing cell-instructive cues, could favor the lacking functional recovery. This would lead to a greater host response, preferably increasing myogenesis. The engineered tissues are, however, also in motion, constantly remodeling and changing, similar to the in vivo tissues, creating a dynamic landscape. This poses a great number of complexities that need to be taken into account, such as an increase in oxygen and nutrient consumption, tissue immaturity, and host integration. Furthermore, sustainable vascularization and innervation are highly important parts of the grand puzzle. The gradual passage from a "simple" cell-free scaffold implantation to tissue engineering is often discreet and requires an understanding of biomimetic methods.

**Figure 1:**
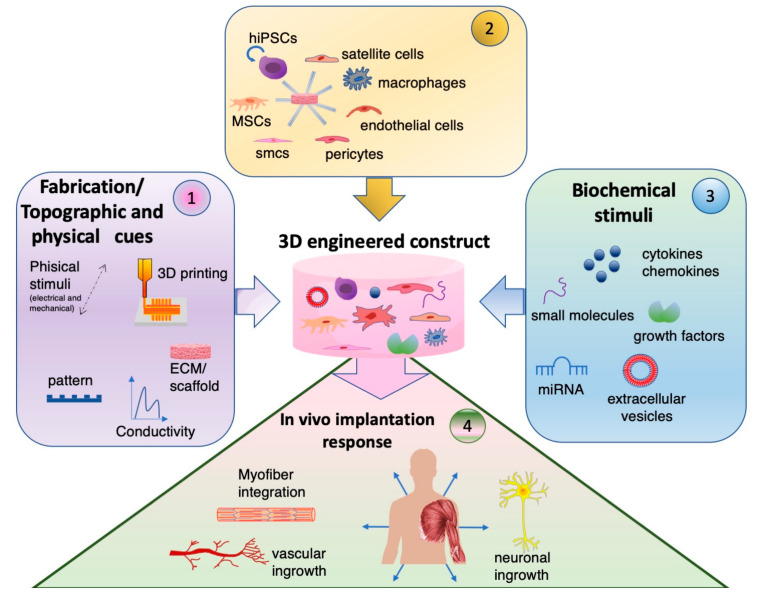
Next stage approach for muscle tissue engineering. The next-generation engineered tissues for muscle regeneration should combine (1) technology cues (fabrication/topographic and physical cues), (2) multiple cell types, and (3) biochemical factors. This complex, multi-cellular and multi-factorial construct is expected to support the de novo formation of the neuro-vascular cell compartment and functional myofiber integration upon in vivo implantation (4).

**Figure 2:**
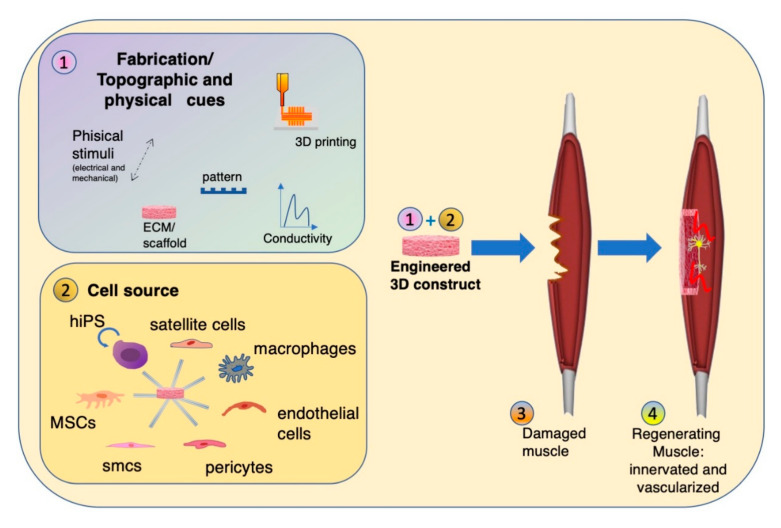
Muscle tissue engineering. (1) Tissue engineering technologies (fabrication/topographic and physical cues) are needed to generate a 3D muscle-like construct together with (2) different cell types: satellite cells, macrophages, endothelial cells, pericytes, smcs, MSCs. The 3D engineered tissue is applied to a damaged muscle (3) in order to induce muscle regeneration (4), including a functional innervated and vascularized muscle.

In this review, we will elucidate the three major aspects of tissue engineering skeletal muscle grafts we identified as crucial strategies for fabricating grafts designed for surgical implantation (Figure 1). The first aspect discussed is scaffold and Extra-Cellular-Matrix (ECM) composition used for the in vitro fabrication. Tunable topographic fiber alignment, the structural orientation created by 3D-printing and electrospinning, and chitosan and gold-nanowire implementation will be discussed. The second aspect explored is the importance of a multicellular environment to create a dynamic and biomimetic cell–cell interaction (Figure 2). Methods of engineering the neuro-vascular compartment, as well as the transition from in vitro muscle-like engineered tissue to a fully functional in vivo organ will be discussed. The main in vitro and in vivo approaches for these first two aspects are reported in synoptic Table 1 and Table 2, respectively.

**Figure 3:**
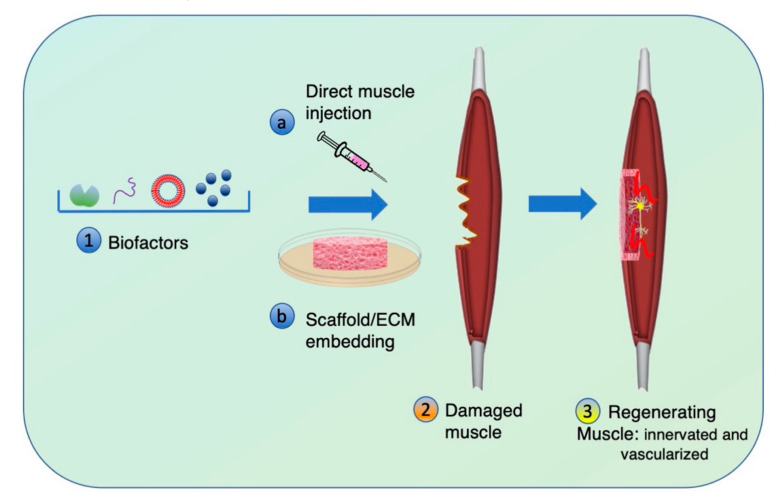
Biochemical stimuli to enhance muscle regeneration. (1) Growth factors, cytokines, small molecules, miRNA are biofactors known to improve the regeneration process (3) of a damaged muscle (2). The biofactors can be delivered to a damaged muscle by (a) direct injection or (b) previous embedding in a scaffold. The factors can be also vehicled by plasmid or virus to enhance the release and possibly achieve innervated and vascularized muscle.

The third aspect covered is the role of biochemical stimuli and their importance in conjunction with the cellular dynamics (Figure 3). Extracellular vesicles have recently gained much appreciation for their modulatory role, and when combined with the ECM, they offer a far greater, positive complexity to the tissue engineering modality. 

We report on the current situation of these topics, discuss recent breakthroughs, and investigate their relevance for future and continued development, with special regard to vascularization and neuronal innervation strategies.

## 2. Scaffold Composition, Topography and Fabrication

### 2.1. Scaffold Composition

The extracellular matrix (ECM) is the net of growth factors, proteins, adhesion molecules present in all tissues; it provides mechanical and chemical support to cells, which in turn give rise to cell–ECM and cell–cell cross talk.

Cell survival, differentiation, functionality and maturation are all therefore also reflection of the ECM, and the choice thereof, ultimately, is paramount for triggering the desired tissue repairing effects [31]. When engineering biological tissues, a scaffold is used to encompass the cells, forming the ECM and the basis of engineered tissue, providing support for the cells, along with a complex environment allowing for cell–cell interaction. It is also possible to create fully personalized scaffolds, seeded with patient derived cells, as was recently demonstrated using extracted pieces of omentum tissue **[32].**

There are a large number of different materials available, and the benefits and disadvantages of the individual ones have already been covered by several extensive reviews and shall not be covered in detail here [33,34,35,36]. In brief terms, the choice of scaffold depends primarily on the desired outcome, fabrication method and cellular inclusion. A variety of scaffolds from natural sources have been described, such as collagen, fibrin, alginate or Matrigel. Additionally, hyaluronic acid (HA) [37,38], gelatin [39] silk fibroin [40], and chitosan [41] find application in muscle tissue engineering. A large variety of artificial scaffolds, in comparison, are manufactured from biodegradable polyesters of polyglycolic acid, polyethylene glycol (PEG), polycaprolactone, poly(lactic-co-glycolic acid), and poly l-lactic acid [42,43].

During scaffold fabrication, the structural properties of the scaffold can be further fine-tuned by functionalizing with RGD [44] or crosslinking with molecules [45] nanoparticles [46,47] and proteins such as laminin [8,48]. The protein fiber length, pore size, orientation and stiffness [49], to name a few, demonstrate the infinite variables to be considered.

A material rapidly gaining interest in additive manufacturing is chitosan. Derived from chitin, found in the shells of shrimp and other crustaceans and produced at a large scale from fungal source, it is a biocompatible, renewable material [50]. The unique intrinsic properties allow for chitosan to be used alone or co-formulated with other materials. In the past it has been widely used for bone and skin tissue engineering; however, exploitation of these properties could allow for extension into skeletal muscle engineering. As a scaffold, it can support smooth muscle differentiation [51] and MSC retention for implantable models [52], demonstrating the positive cellular environment. The electroconductive properties can be readily improved with additives such as calcium silicate [53] and carbon nanotubes [54], offering increased potential for myogenic maturation. In vivo analysis in a mouse dorsal skinfold chamber demonstrated an enhanced angiogenic potential and host-cell ingrowth [55]. When functionalized with RGD, it was shown that a hydrogel containing chitosan provided a better growth environment for not only MSCs but also Endothelial Cells (ECs) and Smooth Muscle Cells (SMCs) [47]. An exciting development was recently described by Guo et al.: chitosan was synthesized as an injectable hydrogel with encompassed C2C12 myoblasts and Human Umbilical Vein Endothelial Cells (HUVEC). The cells were released in a linear-like profile, and the released myoblasts retained continuous proliferation ability, further demonstrating the potential regenerative ability of chitosan scaffolds [23]. Chitosan manufacturing depends on a variety of variables and a magnitude of factors such as pH, water content, molecular size and porosity can affect the cellular compatibility. This requires a good understanding of the basic molecular characteristics. So far, it is seen as non-immunogenic, non-toxic, biodegradable and even boasts anti-microbial and anti-fungal properties [56]. These, however, are also limitations, as further research is greatly needed to confirm these ideas and consolidate them.

An acellular scaffold, as the name implies, is cell-free and relies on the host regeneration response. These cell-free scaffolds can be implanted into muscle defects, aiming directly for host-cell invasion and improvement of function. Certainly, the greatest benefit from all these scaffolds is surely the availability and facile usage. Scaffolds can be made in advance and packaged so only to be opened upon need. Furthermore, a lack of cells also means better biocompatibility, and thus broader application fields. Collagen-GAG scaffolds, to name one of many, present an interesting take on this field, shown to increase hypertrophy and promote gene expression in long term animal models [22].

Another exciting acellular concept is that of decellularized tissues [9]. They provide a complex pre-existing ECM architecture, naturally mimicking the in vivo environment. Elegant works describe the paramount role that decellularized muscle tissues, applied in a muscle defect possess in providing the topographical parameters [57] together with the cytokines that enhance tissue regeneration [58], although with only partial functional recovery. The limitations are mainly focused on the decellularizing technique used, aimed at maintaining the pre-existing ECM and especially the protein binding sites. During decellularization, changes in the original architecture are inevitable and are not yet fully investigated. Implantation of decellularized ECM grafts, has however already been tested in human clinical trials, and documented to lead to increased angiogenesis and perivascular stem cell invasion [59]. In this study, decellularized urinary bladder ECM was 5 male human patients with varying VML injuries ranging from 58 to 90% tissue deficit underwent implantation, and 3 of 5 showed a functional improvement after 6 months.

### 2.2. Tunable Factors

During myogenesis ex vivo, maturation is a summary of a magnitude of cues and stimuli, such as the alignment and topography of the substrate, but also achieved by electrically [60] and physically [61] training the myoblasts. Electrical stimulation for example, is known to increase the glycolytic and fatty acid metabolic flux [62]. During tissue maturation, a more uniform electrical transmission of engineered tissues is achieved with electrically conductive materials. This can be improved by adding conductive materials to the scaffolds during fabrication, such as graphene oxide [63] or nanoparticles [64]. A further example are gold nanowires, mixed with a collagen-based bioink and electrically aligned during 3D printing to provide topographical cues to the cells. In comparison, mechanical loading increases hypertrophy and force production, as has been demonstrated many times. Ref. [65] Interestingly, mechanical loading was recently shown to significantly increase insulin-like growth factor-1 and matrix metalloproteinase-2 messenger RNA expression and significantly downregulate the levels of the atrophic gene muscle atrophy F-box [16]. Loading the cells through stretching in a controlled manner can even lead to the differentiation of mesenchymal stem cells [66]. A very exciting technique was presented by Zhu et al. Engineered ECM scaffolds with sacrificial microchannels were implanted subcutaneously into rats for 4 weeks, during which time newly formed fibrous tissue occupied the inter-fiber space. Afterwards the explanted scaffolds were decellularized, then implanted into a VML rat model. After 1 month, a significantly higher number of neo-muscle fibers, new blood vessels and nerve migration could be distinguished. [26]. Maturation of engineered skeletal muscle tissue through external stimuli is not a new concept and a great deal of research has already been achieved on this topic. Modern approaches surely combine multiple stimuli [8], and the focus has shifted from the first approaches on how to stimulate the tissue, to more complete methods of cellular dynamics.

#### Topography

The orientational and physical cues required by differentiating and maturing myoblasts have long been known to not only arise from the scaffold but also from the forces working on it. Micropatterning of substrates have been shown to be key regulators for controlling the size and alignment of forming myotubes during myogenesis [67,68,69]. A good example of the effect substrate patterning can have was recently shown with vascular smooth muscle cells (vSMC). The vSMC changed their morphology and improved differentiation when exposed to the intracellular force reduction caused by a micro-grooved substrate surface [70]. This highlights the effect, not only on skeletal muscle cells, but also on the supportive cells found in healthy, native muscle tissue. A further interesting take was combining this with a thermo-responsive surface. Nanopatterned cell sheets were created and formed around shapes to recreate the curvature of specific muscles, such as the abdominal muscles **[71].** Nakayama et al. presented a spatially patterned engineered muscle with endothelial cell co-culture, showing highly organized myofibers and microvasculature with functional improvement in vascular perfusion and cellular retention in comparison to engineered muscle from randomly oriented scaffolds, when implanted in an animal model [72]. These fundamental studies help further our understanding of myogenesis and muscle development, crucial for further improving and advancing the field of tissue engineering. However, it seems to only scratch the surface of complexity expected during the engineering of such complex tissues. Additionally, the relevance of a 2D-micropatterned surface during the fabrication process of a thick, 3D tissue, especially on the cells of the opposite surface or in the interior, is questionable.

To try and overcome this distinct limitation, in larger and thicker tissues, one method might be to modulate the individual ECM fibers, patterning them. Carnes et al. described a process of drying fibrin and thrombin microthreads in an acidic environment, thus etching the surface leading to grooving thereof. After cell seeding, enhanced alignment and actin stress fiber organization was noted. [73] Similar findings were demonstrated only when a 3D-printed PCL-based scaffold was stretched under defined conditions, leading to greater surface roughness and protein absorbability. Cultured myoblasts on the surface aligned along this pattern and were more elongated [12]. Longitudinal topography induced by stretching can even be done with very long fibers, up to 1m with diameters of 0.2–3mm [74]. The importance of fiber alignment was recently demonstrated in vivo, after autografts were implanted into an animal model at varying degrees of rotation. Aligned grafts had increased myoblast determination protein 1 (MyoD) and Paired Box 7 (Pax7) gene expression, combined with greater torque recovery and mass after the investigation period [25]. Spatial cues during cell culture and cellular interaction can be further investigated on a micro scale. Kankala et al. were able to show an augmented myogenic differentiation and cell elongation when proliferating skeletal myoblasts were cultured on porous microspheres created using microfluidics [75]. Similarly, gelatin bubbles created using a microfluidic device could well sustain myoblasts, offering a 3D culture environment [13]. As a whole, substrate patterning is a valuable method to study myogenesis and muscle development. This can help to further our understanding not only of myoblast behavior, but also of myofiber-vessel and myofiber-nerve interactions [76]. However, during the fabrication process of tissue-engineered skeletal muscle, the question arises as to how to implement these findings in a relevant manner, especially at a clinically relevant thickness. Scaffold composition and cellular maturation techniques potentially warrant greater efforts, with micropatterning taking a smaller role.

### 2.3. Fabrication Methods

#### 2.3.1. 3D-Bioprinting

As an additive manufacturing technique, 3D-printing is gaining massive interest and the initial cost estimates are dropping due to expiring patents for printers and techniques and overall improving knowledge [77]. 3D-printing, as a field of engineering, allows for the creation of complex 3D structures previously not thought possible, and the advantages over conventional tissue engineering strategies seem immense at first sight. Foremost among these is the achievement of scaffold anisotropy, known for many years to play an immense role in cell alignment and maturation [78].

In vivo skeletal muscle is aligned in a hierarchical fashion with bundles of highly aligned fibers, and a network of vasculature and innervation. Cell alignment is further achieved by directly printing cells in a specific location, in conjunction with other cells or materials.

Furthermore, biofabricating scaffolds is often done using polydimethylsiloxane (PDMS), as a commonly used, two-component molding material. It boasts a high transparency, biocompatibility and gas permeability; however, it can absorb hydrophobic materials, thus potentially having an effect on the cellular interaction. Often, the creation of prototypes is a long process, increasing manufacturing costs. 3D-bioprinting can greatly speed up this process, especially with the range of biocompatible materials available.

The choice of ink, specifically called "bioink", depends on the desired tissue to be created and the biocompatibility, rheological properties and working temperature must be, at minimum, cell compatible, if not cell specific. A great deal of research has gone into finding precise compositions and printing procedures. This topic is complex, and merits a review of its own, so we will kindly refer to other publications covering this area [79].

When approaching 3D-printing of skeletal muscle, the immortalized C2C12 myoblast line is often used a proof of principle. A fundamental goal is to achieve tissue maturation and can be measured by the alignment of the myoblasts and up-regulation of myogenic genes such as myosin heavy chain (MyHC) and myogenin (MyOG) [10].

An exciting development was recently described by Guo et al.: chitosan was synthesized as an injectable hydrogel with encompassed C2C12 myoblasts and HUVEC. The cells were released in a linear-like profile and the released myoblasts retained continuous proliferation ability, further demonstrating the potential regenerative ability of chitosan scaffolds [39].

An exciting concept to be mentioned is that of photocrosslinkable tissue-specific bioink, created by means of decellularization, and subsequently 3D-printed together with induced pluripotent derived cells [80]. This can be taken a step further, co-culturing vascular cells and muscle cells in the same construct, aiming to create a more realistic, patient-specific, final tissue [24]. High-throughput models help our understanding of such muscle cell–vascular cell interaction and has advanced with the reproducible printing of contracting smooth muscle cells [81,82]. Slight additions to the bioink composition, such as the addition of oxygen-generating particles, offer the possibility of improving the metabolic activity of cells [83].

There are certainly several challenges yet to be overcome in the field of 3D-bioprinting. Extending the method of 3D-printing into soft, biological materials poses very different challenges. Problems such as the caving of hollow structures or the need for support structures are basic considerations. Size also matters, as there is a certain limit to the printing resolution. It is currently around 10 times higher than that needed to achieve a resolution comparable to the cell size [79]. The printing speed, temperature, extrusion force and pH are further variables that impose stresses onto the printed cells, making successful and reproducible printing of sensitive cells very difficult. Despite very few reports [84], there have not been any ground breaking advances achieved in multicellular and multi-material printing, due to the inherent complexity and physical printer boundaries. Substrate stiffness as a prime example, set as the optimal range for skeletal muscle cells, might negatively impact the migration and elongation of schwann cells for instance [85], or MSCs during vasculogenesis [86]. Finally, more so than in conventional fabrication methods, the maximum tissue thickness achievable is a stark limiting factor. Due to the longer fabrication time, during which there is a lack of oxygen and nutrient transport, sensitive cells are affected.

#### 2.3.2. Electrospinning

Electrospinning is, unlike 3D-bioprinting, not classed as an additive manufacturing technique. It shares some basic common principles, but follows, however, a dynamic and chaotic fiber deposition. In short, an electric field is applied between the nozzle tip of the printing machine containing the scaffold solution and the grounded collector. An electrified liquid jet is formed, drawn through the air, depositing a non-woven mesh of randomly orientated fibers on the collector [87]. The greatest advantage over 3D-bioprinting is the ability to create ultrafine fibers with a large area-to-volume ratio. The environment therefore extends a full magnitude lower, from the micro- to the nanoscale [88,89]. By changing the voltage applied, components in the solution and distance and rotation of the collector plate, many variables can be altered. A variety of approaches have been discussed as to how to fabricate the fibers in a more controlled and organized manor. It is possible to divide the methods into a variety of categories, depending on the forces involved [90]. By rotating the collector at a high speed, the fibers are stretched and align in accordance with the rotational speed. By applying an external electric field, the alignment of the fibers can be manipulated due to the distributed electric charges along the jet. A further method is to apply a magnetic field, and thus stretch the charged fibers onto the collector. Techniques like these allow for the control of the alignment of fibers created, to a certain extent [91]. Recent advancements have even demonstrated the formation of tubular scaffolds, with a bilayer construction up to 7 mm long [92].

Scaffolds created by electrospinning have also been widely used as a method for examining cell migration and organization [93,94,95]. Furthermore, it has been proven in multiple studies that the physiochemical properties of the ECM scaffolds can promote muscle cell orientation and maturation [96] and support satellite cell growth and myogenic protein expression, [97] as well as the differentiation of mesenchymal stem cells [98,99,100]. Apsite et al. developed a technique whereby a bilayer of electrospun fibers could be induced to self-fold and incapsulate myoblasts. The seeded myoblasts orientated themselves along the anisotropic fibers and formed myotubes. [11] To investigate cellular dynamics on electrospun ECM, it is generally a two-step process. Firstly, the scaffolds are produced and secondly, the cells are seeded onto the scaffold. A great disadvantage, therefore, is the challenge of equally distributing cells during seeding, as well as the poor cellular infiltration of the micro- and nano-fibrillary meshes [101]. Recent publications have developed methods how to incorporate cells into the scaffold directly during the fabrication process, thus shortening the scaffold creation to a single step procedure [102]. By modifying certain parameters, cell survival was documented to be at 90% [103]. Guo et al. showed that by myogenically inducing C2C12 cells, electrospun into fibrin microfiber bundles, compared to uninduced cells, a statistical increase the myotube length, diameter and myotube-associated nuclei was documented [104]. Similarly, research by Gilbert-Honick further supports the advantages of electrospun fibrin microfiber scaffolds. Acellular scaffolds implanted in a mouse VML model could fully recover muscle contractile function after 2 and 4 weeks. When seeded with C2C12 myoblasts, no statistical difference could be found; however, the recovery was quicker [105]. Very recently, the same authors successfully modified the electrospun bundles with the heparan sulfate agrin. After 4 weeks, a higher density of regenerating myofibers was noted, with increased neuromuscular junctions, vascular and neural infiltration, as well as an increase in nuclear yes-associated protein [106]. This demonstrates the exciting potential of electrospinning and further research, especially on multicellular constructs and pharmacological modification, is definitely warranted. The possible effect of the electromagnetic field and mechanical forces acting on the cells during electrospinning are also important fields of future research, investigating how they could alter specific protein channels or cause cell membrane distortion. Furthermore, similar to 3D-bioprinting, the extrusion stress during fabrication may prove to be detrimental to sensitive cell types.

In summary, tissue engineering has profited greatly by the advances of ECM scaffold composition and fabrication methods (Figure 2). The limitations generally found in conventional methods, however, remain, with tissue thickness, vascularization and cell viability defining the outcome and size of the end result [107]. Combinations of methods, such as electrospinning and 3D-bioprinting [27,108], while taking multicellular environment, cellular maturation techniques and ECM topography into account, are currently the most advanced.

## 3. Multicellular Environment for Muscle Tissue Engineering

Skeletal muscle is a complex structure with a meticulous architecture and many different cell types supporting the muscle fibers in their development and function. The previously described fabrication techniques, combined with the proper biomaterial choice and topographical cue, help in mimicking the ECM composition and architecture of the native tissue. Nevertheless, mandatory for developing new methods in tissue engineering is still the basic understanding of cell–cell interaction and of the wide diversity of cell types in skeletal muscle. Liu et al. recently described a new cell type, called teno-muscular cells (TMCs) [109], belonging to the class of rapidly adhering cells, which promote the myogenic differentiation of myogenic cell progenitors. The recent identification of TMCs only further underlines how much is yet to be discovered in this field. The dawn of stem cells has led to great advancements, with their origins ranging from embryonic or amniotic [110] or induced [111] pluripotent stem cells (PSCs) to adult multipotent blood vessel-associated mesangioblasts, bone marrow or adipose (ASCs) derived mesenchymal stem cells (MSCs) [112] or, last but not least, satellite cells. A multicellular environment is truer to native tissue, and the cell–cell interaction launches cascades of signaling molecules and growth factors in a supportive role, also in order to promote in vivo myogenesis, tissue maintenance, vascularization, innervation and host-tissue integration (Figure 2).

### 3.1. Stem Cells

Among the muscle stem cell candidates, muscle resident satellite cells are the ones with the highest myogenic repair potential. Upon damage, the quiescent resident muscle stem cells, namely satellite cells, located between the basal lamina and sarcolemma of adult skeletal muscle fibers, become activated, differentiate toward myoblasts together with mesodermic cells [113] and undergo fusion into myofibers. Post-muscle-growth hypoxia spurs vascular endothelial growth factor (VEGF) release, recruiting perivascular cells. Recently, it has been shown that by modifying the Notch and platelet-derived growth factor (PDGF) pathways of satellite cells, treated cells acquired perivascular cell features and trans-endothelial migration ability [114], further extending our understanding of the plasticity of a multi-cellular environment. An exciting breakthrough in patient-specific treatments was the induction of pluripotent stem cells several years ago. Other fields such as cardiac muscle repair and bone repair have seen significant advancements, yet until date there are very few examples of using iPSCs to engineer skeletal muscle tissue [115]. This could be in part due to the relative ease of isolation of sufficient amounts of skeletal myoblasts for animal and proof-of-concept studies. In 3D culture conditions, Rao et al. [116] could reproducibly form functional skeletal muscle tissues containing aligned myotubes, and combined with the culture time of 1 month, only improving structural and molecular maturation.

Recently, Pax-7 positive satellite cells with a robust myogenic potential were successfully differentiated by mimicking embryonic key signaling events leading to muscle formation. Unlike other differentiation protocols, the one developed by Chal et al. has the unique feature of being a direct one, which avoids further genetic modifications or cell sorting [117]. A further benefit of integrating iPSCs into the engineering approach is certainly the derivation of multiple cell types from the same patient-derived iPSCs cell line. Great focus has also gone into harnessing the vascularization and innervation of the iPSCs [118]. This could mean the generation of muscle fibers, premature vascularization and innervation simultaneously, either by co-culturing terminally differentiated cells, or their progenitor cells. The latter, recently described by Colunge et al., investigated a common vascular progenitor, dubbed "MesoT" [111]. These progenitor cells were shown to contribute to neovascularization of damaged tissue, while also retaining the potential to differentiate into endothelium, pericytes, smooth muscle cells and pericytes.

The surrounding connective tissue also plays a major role in orchestrating muscle morphogenesis and influences muscle precursors, as can be seen from an embryological point of view [119]. There is a growing trend and understanding that a multicellular approach is required to create a complete engineered tissue. With a more thorough understanding of the dynamic cellular interaction pathways, slowly but surely, we are advancing away from single cell engineered constructs and advancing towards a more complex and natural tissue architecture. Different co-culture approaches of muscle stem cells with main progenitor and differentiated cells will be described in the next paragraphs as well as the specific cell interaction with immune cells.

### 3.2. Co-Culture with Adipose Tissue-Derived Stem Cells

ASCs are a readily available cell source, easily maintained and expanded ex vivo. A myoblast-seeded scaffold, co-cultured with human endothelial cells and ASCs was implanted in a Volumetric Muscle Loss (VML) model. After 2 weeks, the implant readily vascularized with the host vasculature and showed red blood cell perfusion [105]. This increase in vasculature is likely not entirely due to a single effect, but to a multifactorial paracrine action of ASCs. When modified to overexpress Hepatocyte Growth Factor (HGF), a superior blood flow restoration, tissue vascularization and innervation, and fibrosis reduction after transplantation was found in a mouse hind limb model [120].

Kang et al. [121] recently described a protocol to differentiate ASCs into cells with functional properties of mature Schwann cells, thus elaborating on the astounding potential of ASCs in a combined neuromuscular regenerative approach and possible applications for a multicellular biomimetic environment.

ASCs are also greatly affected by the culture environment [122]; for example, when seeded onto 3D fibrin fibers they commence myogenic differentiation [123] and can increase in vivo muscle reconstruction following VML defect [124]. This dependency on scaffold fiber orientation and also stiffness [125,126] could also be seen when ASCs were co-cultured with myocytes, and both cell types underwent stiffness-mediated migration. Neural cells in the same model, however, did not, and were seen on softer regions of the hydrogel model for prolonged culture time [127]. Additionally, the environmental factors such as blood flow partake in cell differentiation and fate, like An et al. [128] were able to show in a rat groin, by connecting a bioreactor to an epigastric adipofascial flap and the epigastric vessels, leading to trans-differentiation of adipose tissue into muscle tissue. Models like this help further our understanding of individual growth factors contributing to vascularization and tissue maintenance.

One possibility for taking advantage of growth factors in a more complex environment is in stromal vascular fraction (SVF). Adipose tissue, usually in the form of lipoaspirate undergoes centrifugation and enzymatic digestion, removing the fibrous tissue and adipocytes. The remaining heterogenous cell population contains several cell types, including ASCs, endothelial progenitor cells and pericytes. There are several clinical studies involving SVF currently underway [129], and the benefits for skin treatments, for example in post radiation injuries, burns or chronic ulcers, have already been proven [130,131]. This cell mixture can be used in a variety of ways, like the co-culture with skeletal myoblasts to form 3D-engineered muscle-like tissues [132] or the culture as a cell sheet [133], and is termed by some to be a relatively simple, cost-efficient and autologous way to promote in vivo vascularization. A valid argument as to why SVF is so promising for vascular tissue engineering is certainly the high pericyte content. Pericytes are thought to have a complex secretory ability [134], not only restricted to the maintenance of small caliber vessels, but also in regulating myogenic differentiation and angiogenic potential [135].

Although SVF, as a heterogeneous and autologous cell source, shows a great translational potential, its clinical use in standard treatments might still be questionable since not all patients may undergo an additional invasive liposuction procedure, and its composition is still largely donor-dependent. Until superior cotrol of SVF cell composition has been achieved, muscle stem cells could be combined with a predifined amount of vascular cells and neuronal cells.

### 3.3. Co-Culture with Vascular Cells (Pre-Vascularization Approach)

A grand challenge in the field of tissue engineering is the creation and maintenance of tissues of a functionally relevant thickness. Above the critical diffusion limit, the tissue core becomes necrotic. In vivo, the availability of oxygen and nutrients in skeletal muscle is paramount [136], and the ability to maintain perfusion levels under strain [137] and rapidly increase vascular perfusion in response to exercise and hormonal cues such as insulin [138] are necessities for adequate function. Surprisingly, the limiting factor of muscle function is often of cardiovascular and respiratory nature. On a microvascular level, our knowledge is changing, giving a much greater role to the blood–myocyte interface, whereby the erythrocyte dynamics and distribution are crucial [139]. For instance, in vitro, the capillary sprouting of HUVEC was achieved using the conditioned medium by C2C12-VEGF transfected cell sheets [15]. When developing engineered tissue, be it for direct clinical translation or as a model to study physiology or disease, vascularization or perfusion of the tissue thus plays an important role [140]. When approaching vascularization of engineered tissues, two prevalent methods are currently applied: pre-vascularized tissues in vitro and self-organization [141]. The former method often involves preforming channels in a custom scaffold. Osaki and colleagues built two vessel microchannels with HUVEC in a collagen gel that surround a C2C12 muscle fiber bundle. The endothelial cell sprouting was enhanced when optical stimulation induced C2C12 contraction [14]. A common limitation of this approach is the size of the vessels that can be reproduced in vitro. A well-connected network of capillary-sized vessels is difficult to engineer. A further approach investigated is to allow for the self-organization of organoids. The idea of self-organizing structures is of great interest, not only from a regenerative point of view, but also regarding the macroscale and massive complexity of the blood vessels in human tissue. Self-organized structures might indeed have the benefit of having a greater sensitivity to angiogenic cues, allowing for faster integration into the host circulation system and specifying into functional arteries, arterioles and veins [142,143]. Organoids can also be patient specific, created from differentiated human induced pluripotent stem cells (iPSCs), and biomanufactured at a high density, furthermore using novels methods such as 3D bioprinting to form perfusable channels within the high-density tissue [140]. A biodegradable scaffold can also be made by technique of electrospinning, forming fibers of capillary size and growing cells to confluency, such as HUVECs, around these fibers [144].

A combination pre-vascularization and self-organizing of vascular structures was investigated in a two-stage approach. Compared to a simple co-culture of human muscle cells with endothelial cells in 3D, a one-week-old engineered tissue containing differentiated myotubes was coated with a fibrin gel containing endothelial cells. This two-stage approach led to an improved vascular density, with better interconnected and longer vascular networks [28]. After the physical creation of a 3D tissue, in vivo testing and the question of host acceptability and integration certainly belongs to a complete experimental read-out. Decellularized vessel conduits, do readily recellularize and are supported by a network of microvasculature initially formed by CD34+/CD31+ cells and later maintained by CD34-/CD31+ cells [145].

### 3.4. Nerve Transfer Approach and Co-Culture with Neural Cells

The neuromuscular junctions in humans are among the smallest known and are particularly susceptible to pathological processes [146,147]. A major challenge is to update and further develop established working models to include a sustainable neurological integration [148], as a timely innervation prevents atrophy. Current treatment options to repair damaged nerves include direct repair (nerve grafting), end-to-side neuroraphy and neurotization (nerve transfer) operations. These pose a variety of predicaments, such as lengthy operations, as well as an unsure success rate and therefore a great need for alternative treatment strategies exists. Novakova et al. [21] demonstrated a very complete model, by combining skeletal muscle units, with so-called engineered neural conduits. The proposed neural conduits were formed by wrapping 4 monolayers of tendon lineage differentiated bone marrow stromal cells around a nylon tube. After devitalization at −80 °C, the conduits could be stored and just before implantation the nylon tube was removed. In an ovine model of peroneus tertius VML, the nerve conduit was placed in between a re-routed distal branch of the peroneal nerve and the implanted tissue engineered skeletal muscle unit, to act as a bridge to guide the nerve during innervation. After 3 months, the repair group demonstrated undistinguishable force and muscle mass when compared to the control group. Furthermore, in 75% of the animals in the intervention group, the re-routed peroneal nerve could be stimulated. By creating a separate nerve conduit from a different material than the skeletal muscle unit, certainly overcomes the different material limitations that favor either myogenic or neurogenic cells. It is open to debate whether the implanted nerve conduit actually favored neuronal ingrowth, or just acted as a barrier, shielding the nerve from the post-operative inflammatory microenvironment for sufficient time.

Interestingly, co-culturing myoblasts with neural cells leads to improved contractile force generation, thus improving the functional outcome [17]. This further underlines the necessity of a multicellular tissue engineering approach in supporting functional recovery. Very interesting work performed in vitro demonstrated that co-cultured muscle cells and iPSC motoneurons derived in a 3D polydimethilsyloxane (PDMS) scaffold could be used to study neuromuscular junction (NMJ) formation in a healthy and in neuronal disease model [18]. The same co-culture method (with iPSCs or embryonic stem cells) in a microfluidic device created an NMJ tissue-engineered model. This represents a very useful tool to unravel not only the mechanisms leading to NMJ degeneration and repair but also a patient-specific drug-screening platform. A recent study transferred the femoral nerve to a muscle graft in an abdominal wall defect model, showing an increase in the formation of neuromuscular junction and axonal penetration compared to the non-neurotized grafts [149]. Skeletal muscle cells secrete proteins that serve as a communication link to the brain, by enhancing neural differentiation [150]. At the same time, too many neural cells seem to inhibit myotube formation, and a ratio of 300:1 (human muscle progenitor cells: human neural stem cells) was deemed to be optimal by Kim and colleagues [20]. Furthermore, a higher cell viability was present in the co-culture constructs when compared to a muscle cell mono-culture. This was combined with an increase in myotube density and length, as well as the presence of glial cells and neuros in contact with acetylcholine receptor clusters (neuromuscular junctions). In a 40% VML rodent model, implantation of the co-culture 3D construct led to a full force recovery when compared to the sham operation group, whereas the muscle cell mono-culture 3D construct only led to a 70% force recovery after 8 weeks. Interesting findings by Das et al. [19] further support a co-culture environment. When myoblasts and spinal motor neurons were co-cultured on aligned nanofiber scaffolds (providing topographical cue), a pro-regenerative microenvironment formed promoting vascularization and an increase in satellite cells. Similarly, co-cultured constructs were found to have a significantly higher percentage of mature neuromuscular junctions. The limitation of this particular study is certainly the in vivo model chosen. A 20% VML rodent model with implantation time of 1 or 3 weeks is, as previously highlighted, not a sufficient quantitative muscle loss to rule out spontaneous recovery. The results, however, were presented in direct comparison with monoculture scaffold implantation, acellular scaffold implantation or no scaffold implantation after muscle injury. It might therefore be safe to assume a faster satellite cell recruitment in the co-culture group.

### 3.5. Immune Cell–Satellite Cell Interaction

Satellite cells are known to reside in specific niches [151] and aid in muscle regeneration [152], yet lack extended migratory capabilities. Further underlining this cell–cell interactive niche is the necessity for an intact immune system. When satellite cells are embedded in a 3D porous construct and implanted in immunocompetent and immunocompromised mice, after 18 days, the former showed enhanced neovascularization [29]. Immune cells are certainly gaining a foothold in modern tissue engineering to achieve a practical and especially complete, vascularization strategy. Through monocyte paracrine signaling, the cellularity and ECM accumulation in co-culture with derived vascular smooth muscle cells increased [153]. Macrophages, on the other hand, have been directly linked with augmented vessel ingrowth, cell survival, muscle regeneration, and contractile function of muscle [30]. Microvascular fragments from macrophage depleted mice were recently shown to promote the reassembly of these fragments into microvascular networks to a lesser degree [154]. In response to environmental cues, macrophages can also switch to a pro-regenerative M2 phenotype [155]. Similarly, in a transgene mouse model with Duchenne muscular dystrophy, overexpression of regulatory T-cells of the muscle type (muscle-Tregs) saw enhanced accumulation in hindlimb muscles and improved regeneration thereof [156].

## 4. Biochemical Stimuli to Adjuvate Muscle Regeneration

In addition to the appropriate biomaterial and cell type, adequate stimulation strategies are indispensable for endorsing muscle regeneration. Damaged tissue and cells are highly responsive to biophysical (mechanical properties of the biomaterial—stiffness, porosity—, electrical properties, or the combination of the two) and biochemical stimuli. A comprehensive review on the biophysical stimuli has been presented by Somers et al. [157].

In this section, we mainly focus on the biochemical stimuli, starting from the endogenous factors secreted by the muscle cells themselves, to the exogenous factors synthetically produced or derived from other cells, such as the extracellular vesicles. Figure 3 shows how the biochemical stimuli can be applied in muscle regeneration.

### 4.1. Soluble Factors: Growth Factors and Small Molecules

After damage, the muscle cells specifically secreted myokines that influence inflammatory and metabolic processes in an endocrine or paracrine manner [158,159]. Among these, Interleukin (IL)-6 plays a dual role as pleiotropic myokine that influences satellite cell activation and differentiation [160], but also a pro-inflammatory cytokine, not only in muscle, but also in other tissues [161]. IL-6 also downregulates the pro inflammatory cytokine TNF-α and at the same time elevates the anti-inflammatory cytokines IL-10 and IL-1 [158]. Additional chemokines such as CCL17, CCL2 and growth factors (namely, fibroblast growth factor (FGF), HGF, insulin growth factor (IGF-I), tumor necrosis factor-α (TNF-α), VEGF, transforming growth factor β 1 (TGF-β1), and PDGF) are released into the intercellular space to modulate signals for muscle repairing. 

Soluble factors can be delivered following injection or using scaffold-based delivery system. The first approach to trigger muscle regeneration is the direct injection of these factors in the damaged muscles. It is well known that PDGF, HGF, FGF activate satellite cells in damaged muscle. There are good examples of muscle cell stimulation after growth factor injection in both models of Duchenne muscular dystrophy (mdx mouse) [162] and of damaged tibialis anterior (in mice and rats) [163,164].

By simultaneously targeting angiogenesis and myogenesis, in the context of ischemic muscle injury, it was shown that the delivery of VEGF in gel with IGF-I or VEGF in transfected C2C12 enhanced the muscle regenerative process, namely angiogenesis, reinnervation, and myogenesis, at the same time [15,165]. Toward this combined approach, platelet-rich plasma (PRP) has been evaluated as possible alternative stimuli [166]. PRP contains autologous growth factors able to trigger skeletal muscle regeneration such as IGF-1, HGF, FGF-2, TGFβ-1, TNF-α, PDGF, and prostaglandins (PG) [167].

In the past, autologous PRP has been considered a safe and efficient product to repair musculoskeletal lesions with sports-related injuries [168]. More recent studies in vitro demonstrated that PRP decrease collagen synthesis and his efficacy started to be controversial [169]. If PRP is associated with TGF-β1 neutralizing antibodies, muscle fibrosis is reduced and regeneration is promoted [170]. However, a randomized clinical trial with muscle injured patients showed no positive effect in time of recovery and reduction in reinjury rate after PRP treatment, as compared with placebo injections [171]. PRP gave also allergic reaction when injected in tibia cyst of a young patient [172]. Section 4.2 will describe small vesicles produced by cells that are full of different biomolecules and could be a good alternative to PRP use.

When growth factors (soluble molecules) are directly injected, the blood stream rapid clearance represents the main cause for the loss of molecule bio-activity. As an alternative, the genetic vehicles enable the in situ synthesis of growth factors within the injured muscle. Toward this aim, ASCs overexpressing HGF showed angiogenic and neuroprotective effects in a murine model of hind limb ischemia model [120]. Ye and collaborators demonstrated that injecting a recombinant adeno-associated virus encoding for IGF-1A in immobilized soleus muscles, the muscle regeneration was accelerated [173]. Using the reprograming strategies, the tunable enhancement of chondrogenic and myogenic differentiation was obtained when a plasmid encoding the transcription factors SOX9 and MYOD were entrapped in fibrin hydrogel [174]. Although the gene can be delivered to the target tissue, a major limitation of this approach is the lack of control of the gene delivery process. 

Inclusion of molecules in scaffolds represents a promising approach that avoid the rapid blood clearance. As an example, to enhance angiogenesis, 5-ethyl-5-(hydroxymethyl)-β,β-dimethyl-1,3-dioxane-2-ethanol diacrylate (EHD) was crosslinked with PEG and subsequently used to deliver a plasmid encoding IGF-I GFP to human myoblasts cells in vitro. The different porous materials tuned the plasmid delivery [175].

Collagen–gelatin scaffold embedded with FGF-2-encoding plasmids and adenoviral vectors induced higher neoangiogenesis and muscle regeneration in a rat muscle defect model in respect to FGF-2 protein alone [176]. Tuning the molecular weight of alginate hydrogel, researchers developed a method able to maintain the delivery of lentiviral vectors up to two months in a hindlimb murine model [177]. Although the increasing expertise in biomaterials and gene delivery, to date no clinical studies have been reported using this approach for muscle regeneration. The limited knowledge of the expression level of the transgene during time in the scaffold, the transfection efficiency, and the safety are still the major unresolved aspects. Besides the inclusion of genes encoding the factor of interest, biomolecules can be linked to the scaffold. Different methods are employed, from covalent bonding, to physical entrapment, or surface adsorption according to the physicochemical properties of both the scaffold and the factors (for a comprehensive review on this matter, see [178]). Natural biomaterials such as fibronectin, laminin, collagen, elastin, the glycosaminoglycans heparin sulphate, chondroitin sulphate, and hyaluronic acid, or a variety of synthetic hydrogels have been used as extracellular matrix-mimicking materials to enhance skeletal myogenic proteins [179] or immobilize factors such as VEGF to induce local angiogenesis [180,181].

In a recent work, IGF-1 and VEGF were embedded in a scaffold made of alginate hydrogel for muscle functional recovery in a rabbit model of craniofacial defect [182]. The functional improvement was not attributed to structural neuromuscular junction connection, but appeared to be more related to increased neurotransmitter release or enhanced postsynaptic responses. In a sciatic nerve defect model, an acellular nerve scaffold with VEGF-heparin release system allowed the achievement of early vascularization and blood supply in the nerve graft area. The early vascularization in the area of the damaged nerve promoted not only the nerve repair, but also the restoration of normal morphological structure and function of peripheral nerves [183]. Rybalko and colleagues, in a model of ischemia/reperfusion, vehicled SDF-1α/IGF-I in a PEG fibrin hydrogel obtaining neovasculogenesis and force recovery. Here, the concerted action of SDF-1α, which sustained stem cell recruitment to the site of muscle injury, and of IGF-I, which promoted cell differentiation, was paramount to reaching the functional muscle [184].

Using a novel poly ethylene glycol PEGylated fibrin gel matrix (PEG-Fib), we incorporated SDF-1α alone (PEG-Fib/SDF-1α) or in combination with IGF-I (PEG-Fib/SDF-1α/IGF-I) for controlled release at the site of acute muscle injury. Despite enhanced cell recruitment and revascularization of the regenerating muscle after SDF-1α treatment, functional analysis showed no benefit from PEG-Fib/SDF-1α therapy, while dual delivery of PEG-Fib/SDF-1α/IGF-I resulted in IGF-I-mediated improvement of maximal force recovery and SDF-1α-driven in vivo neovasculogenesis.

While growth factors can retrieve immunological response, small molecules are too small (usually not exceeding 1000 Da) to induce an immune response in the host [185]. Moreover, the delivery of small molecules offers the advantage to reduce the cross-species contamination and the cost of the production in comparison to recombinant protein-based growth factors [186]. For muscle regeneration, sphingosine-1-phosphate (S1P) has been demonstrated to promote cell motility and angiogenesis, in addition to muscle cross sectional area [187,188]. Myoblast de-differentiation was induced using the small molecules BIO (glycogen synthase-3 kinase inhibitor), SB203580 (p38 MAP kinase inhibitor), and lysophosphatidic acid, maintaining the ability to naturally differentiate again [189]. Retinoic acid is another example of a small molecule that is important for differentiation of muscle progenitor cells. It also actively participates in glucose metabolism for muscle cells [190].

### 4.2. Extracellular Vesicles

During the last decade, the deeper understanding of cellular functions and intercellular communications has opened new avenues to improve tissue engineering strategies. Besides myokines, intercellular interactions are arranged by secreted vesicles, defined as extracellular vesicles (EVs). Originally considered cellular debris, EVs are heterogeneous nanoparticles, ranging from 30 to 200 nm, shed via a blebbing or endolysosome process, from all cell types (from prokaryotes to eukaryotes). Accumulating evidence highlights that these nanoparticles, secreted under physiological and pathological conditions, represent shuttles of biological signals among cells and tissues. The cargo derives from their cell of origin and include mRNA, miRNA, genomic and mitochondrial DNA, proteins, lipids and carbohydrates [191].

The use of EVs possesses some important advantages with respect to growth factors or small molecules. First of all, the nanovesicles express surface molecules deputed to cell attachment such as integrins and tetraspanins. Consequently, once the EVs are attached to the cells, they deliver their cargo to other cells. The blood clearance is still a parameter to be tuned, for instance with EV embedding in scaffolds (Figure 3). EVs are a cellular product that does not express immune histocompatibility antigens. So far, many in vivo studies have demonstrated that they do not induce immune rejection but, on the contrary, improve immune modulation [192]. Skeletal muscle cells secrete EVs that contain basic signals for skeletal muscle myogenesis, homeostasis, and development. [193,194,195] However, pioneer works identified proteins and mRNA that have not previously been implicated in muscle regeneration or development but that are involved in cross-communication between muscles and either nerves, vessels, or immune cells [196]. The paracrine signal production is also stimulated by the shape of the biomaterial in which cells are embedded. A recent work demonstrated how porous scaffold instead of compact hydrogel, better promote paracrine responses by enhancing local cell–cell interactions in myoblasts, ameliorating muscle cell migration and proliferation [197].

Human EVs in murine muscle regeneration proved to decrease collagen deposition with low fibrosis and enhanced tissue regeneration [198]. In damaged muscle, although not exclusively, EVs showed immunomodulatory properties [199] with specific ability to polarize macrophages toward the pro-regenerative profile M2 [200]. Investigating the neuronal compartment, it has been shown that EVs derived from the myoblast C2C12 cells stimulate survival and neurite outgrowth of motor neurons by paracrine signals [201]. In a rat model of femoral nerve ligation, EV derived from primary muscle cells significantly influence neuron regeneration [202].

In addition to their secretion of cytokines, chemokines, or growth factors, EVs orchestrate a plethora of signals finally tuned by the surrounding environment [203,204]. Among all the factors vehicled by EVs, deep analysis unveils that non protein-coding RNA molecules, such as miRNA, are also encapsulated and are responsible to regulate gene expression at post-transcriptional stage. Myogenic miRNAs in exosomes, including miR-1, miR-133, and miR-206 exert regeneration effects. For instance, while the local injection of these miRNAs accelerated muscle regeneration in a rat model of skeletal muscle injury [205], the injection of EVs in an ischemic/reperfusion cardiac injury overexpressed the cardioprotective miR-494 [206], stimulating tissue regeneration.

Seminal studies have demonstrated that EVs can be used for regenerative medicine purposes when loaded with a desired cargo such as drug or gene, targeting a specific cell type [207,208,209].

## 5. Conclusions

Guiding the body to regenerate itself seems to be the ultimate goal, as the reality of implanting a piece of a readily functional vascularized and innervated muscle during a short operation and after a short production time is becoming more and more unlikely. It seems that one single biochemical cue, cell type or ECM support alone cannot achieve large-scale muscle regeneration. A future approach should be based on the implantation of an in vitro pre-organized muscle-like tissue with a complex cellular and ECM environment, rich in the key biochemical and topographical cues to support in vivo vascularization, innervation and integration, therefore inducing survival and functionality in the long term (Figure 1). Different materials, while capitalizing on the nano- and micro-fiber alignment, coupled with maturation stimuli such as electricity and strain, provide the backbone for cellular crosstalk. Combining these techniques with different cell types, such as satellite cells, and also with other fully differentiated or progenitor cells in both the vascular and neural lineages are much more effective. In particular, iPSCs and SVF are of great translational and scientific interest, considering that they both possess high muscle regenerative properties with patient specificity. On the one hand, iPSCs are a remarkably tunable biological tool for a wide variety of tissue rebuilding; on the other, SVF is a complex cell population mixture inherently bringing cell–cell interactions and an array of angiogenic, neurogenic and anti-fibrotic cues. In addition to the employment of these cells per se, promising results are conveyed by coupling them with exogeneous stimuli such as EVs, a natural reservoir of biofactors. The EVs can not only be modified according to need, but can also enhance the scaffold bioactivity. However, this is always in conjunction with current physiotherapy strategies, and should no longer be seen as an individual entity, but more in combination, also with mechanical engineering.

## Figures and Tables

**Table 1:** In vitro tissue engineering approaches

In Vitro			
Scaffold	Model	Main Findings	References
**Muscle Compartment**			
PU	PU and dynamically perfused C2C12	3D Polyurethane-based soft porous scaffold functionalized with laminin and fibronectin coating allow better differentiation of C2C12.	Iberite et al. (2020) [8]
PEGDA, colecistic ECM hydrogel.	Hydrogel and C2C12	PEGDA conjugated with porcine cholecystic derived ECM, formed biocompatible hydrogel suitable for growth and maturation of C2C12.	Raj et al. (2020) [9]
PLGA bioprinting	C2C12 in 3D printed scaffold.	PLGA 3D printed scaffold with C2C12 promote myogenesis and upregulate the expressions of myogenic genes (MyHC and MyOG).	Chen et al. (2019) [10]
AA-MA, PLC bilayer scaffold; electrospinning	Self fold bilayer scaffold and C2C12	A bilayer scaffold of AA-MA and aligned PLC seeded with C2C12 form aligned myotubes that contract under electrical stimuli	Apsite et al. (2020) [11]
PCL bioprinting	Stretched 3D printed scaffold and C2C12	3D PCL scaffold used to culture C2C12 lead to better myotube formation when the scaffold is stretched.	Yang et al. (2019) [12]
Gelatin	3D gelatin scaffold and H9c2 cells	Rat H9c2 myotube formation is improved by 3D spherical gelatin bubble-based scaffold compared to 2D gelatin plating.	Mei et al. (2019) [13]
**Vascular Compartment**			
Muscle bundles	Channelrhodopsin-2 C2C12 muscle fiber bundles and collagen HUVEC vascular structures.	Muscle fiber bundle modulate the endothelial cell sprouting. In turn myogenesis was also upregulated by interaction with the vascular cells, improving muscle contraction via angiopoetin-1/neuregulin-1 signaling. Optical stimulation of muscle tissue induces significantly more angiogenic sprouting.	Osaki et al. (2017) [14]
Tetronic-tyramine hydrogel RGD	C2C12-VEGF cell sheets	The conditioned medium of VEGF-transfected C2C12 increases HUVEC sprouting in capillary formation assay.	Lee et al. (2014) [15]
**Neural Compartment**			
Collagen hydrogel	Collagen with differentiated C2C12.	Scaffold with myotubes shows hypertrophy and increased contractile strength after mechanical loading.	Aguilar-Agon et al. (2019) [16]
Collagen and matrigel scaffold.	C2C12 and PC12 in petri dish and 3D Matrigel	Co-culture of muscular and neural cells in a 3D model improve sarcomere formation and contractile activity of differentiated C2C12 in comparison to 2D model.	Arifuzzaman et al. (2019) [17]
Fibrin/Geltrex Hydrogel	Hydrogel with hMPCs and hESC derived motoneuron.	hMPC and human motoneuron co-cultured only when cultured in hydrogel and not in petri dish, show after 3 weeks an increase in myofiber diameter and neuromuscular junction functionality. Calcium imaging proved functional connectivity between motor neuron endplates and muscle fibers.	Bakooshli et al. (2019) [18]
PCL mold	Aligned PCL with C2C12 and E16 Sprague Dawley motor neuron	Co-culture of C2C12 and E16 ameliorate myogenic index of C2C12 myotubes.	Das et al. (2020) [19]
PCL bioprinting	hMPCs co-cultured with hNSCs in PLC contruct	3D construct of hMPCs and hNSCs shows good cell survival, muscle differentiation and NMJs formation.	Kim et al. (2020) [20]

Abbreviations: AA-MA: Anisotropic methacrylated alginate fibers; PEGDA: Poly(ethylene glycol) diacrylate; PLC: Polycaprolactone; PLGA: Poly lactic-co-glycolic acid; PU: Polyurethane.

**Table 2:** In vivo tissue engineering approaches.

In Vivo			
Scaffold	Model	Main Findings	References
**Neuro-Muscular Compartment**			
SMUs and ENC	VML in sheep	3 months post implant, sheep treated with SMU recovered muscle mass and tetanic force production.	Novakova et al. (2020) [21]
Collagen-GAG scaffold	VML in mouse	Increased hypertrophy in treated mouse 2 and 6 weeks post implant. increased running speed on a treadmill after 6 weeks compared to sham mice.	Panayi et al. (2019) [22]
Dex-AT and CECS hydrogel.	VML in rat, injectable hydrogel with C2C12 and HUVEC-GFP.	1 and 4 weeks post treatment, cells were proportionally released over time. Higher myofiber density was present in animals treated with hydrogel and cells when compared with animals treated with hydrogel alone.	Guo et al. (2019) [23]
Porcine muscle ECM sponge and bioink. Descending aorta ECM bioink for bioprinting.	VML in rat and ECM with hSKMs and HUVEC.	3 scaffolds were compared: (1) muscle ECM sponge with hSKMs (2) ECM hydrogel used as bioink with hSKMs (3) gelatin granules mold for muscle and aorta ECM seeded with hSKMs and HUVEC. 4-weeks after implant, scaffold number 3 produced better cell viability, myotube formation, angiogenesis and muscle strength recovery in respect to the other scaffolds.	Choi et al. (2019) [24]
Muscle tissue plug	Rat VML.	3 different alignment (0°,45°,90°) of muscle defect plug were implanted. The best tissue regeneration was achieved with aligned muscle plug (0°): increased expression of myogenic genes 2 weeks after implant; a peak of tetanic torque force and reduced collagen deposition after 12 weeks.	Kim et al. (2020) [25]
Subcutaneous implant of PCL fibers	Rat VML, sciatic nerve and abdominal artery defects.	In vivo implant of depleted PCL ECM allows: (1) muscle tissue regeneration with reduction of collagen deposition; (2) good axon diameter, thickness of myelin sheets; (3) vascular regeneration with good morphology of vascular microchannels	Zhu et al. (2019) [26]
Fibrinogen, gelatin, hyaluronic acid and glycerol bioink in PCL pillar	VML in rat and bioink with hMPCs	8-weeks post implant, muscle strength, vascularization and number of NMJs were higher in comparison with rats treated with bioink without cells (printed and non-printed).	Kim et al. (2018) [27]
PCL bioprinting	VML in rat and scaffold with hMPCs co-cultured with hNSCs.	Analysis were performed at 4 and 8 weeks. Pre-innervated scaffold ameliorated functional muscle regeneration, NMJs formation and reduce fibrotic tissue deposition compared to rat treated with scaffold seeded with hMPCs alone.	Kim et al. (2020) [20]
**Vascular Compartment**			
BAM in fibrin hydrogel	Subcutaneous injection on the fascia of the *latissimus dorsi* muscle of hydrogel and BAM with and without HUVEC-GFP	(1) BAM alone, (2) BAM co-cultured with HUVEC-GFP and (3) BAM with HUVEC in fibrin hydrogel (two-stage approach). 14 days post treatment, myotube length and area, vessel length and branching were evaluated. The number 3 construct gave better results.	Gholobova et al. (2020) [28]
PLLA and PLGA scaffold	Abdominal wall defect in mouse and PLLA/PLGA with satellite and lung microvascular cells.	Scaffolds with satellite cells alone and scaffolds with both cell types were implanted in immunocompetent and immunocompromised mice. 18 days post implantation, the pre-vascularized scaffold inserted in immunocompromised mice showed better neovascularization and myogenesis in respect to the immunocompetent.	Perry et al. (2019) [29]
Fibrinogen hydrogel	VML in rat and hydrogel with BMDMs.	After 15 days, implantation of gel with muscle cells+BMDMs shows increased vascularization, muscle area and muscle strength compared with implantation of gel containing only muscle cells.	Juhas et al. (2018) [30]
Tetronic-tyramine hydrogel RGD	C2C12-VEGF cell sheets	Ischemic model with myoblasts sheets: promoted the formation of capillaries and arterioles in ischemic muscles, attenuated the muscle necrosis and fibrosis progressed by ischemia, and prevented ischemic limb loss.	Lee et al. (2014) [15]

Abbreviations: BAM: Bio Artificial Muscle with human skeletal muscle cells; BMDMs: Bone Marrow Derived Macrophages; CECS: N-carboxyethyl chitosan; Dex-AT: Dextran-graft-aniline tetramer-graft-4; ENC: Engineered Neural Conduit; GAG: Glycosaminoglycans; hMPCs: human Muscle Precursor Cells; hNSCs: human Neural Stem Cells; hSKMs: human Skeletal Muscle Cells; NMJs: Neuromuscular Junction; PLGA: Poly Lactic-co-Glycolic Acid; PLLA: Poly L-Lactic Acid; SMUs: Small Muscle Units.
